# Brain Abscess Due to Lactobacillus Fermentum in an Uncontrolled Diabetic

**DOI:** 10.7759/cureus.26360

**Published:** 2022-06-27

**Authors:** Supraja Achuthanandan, Amit Dhaliwal, Tiffany Lu, Kavita Sharma

**Affiliations:** 1 Internal Medicine, Maimonides Medical Center, Brooklyn, USA; 2 Infectious Diseases, Maimonides Medical Center, Brooklyn, USA

**Keywords:** lactobacillus species, brain abscess mri, immunocompromised brain abscess, abscess, lactobacillus fermentum, diabetes, lactobacillus, brain abscess

## Abstract

Brain abscesses are collections of infectious fluid within the brain parenchyma, with mortality ranging from 15% to 31%. They can result from direct inoculation or via hematogenous spread. Streptococcus and staphylococcus species and Gram-negative bacilli are common bacteria responsible for brain abscesses. In immunocompromised patients, such as those with organ transplants or HIV, brain abscesses can be caused by fungi, mycobacteria, or parasites. Lactobacillus is a very rare cause of brain abscess and has only been observed in a few case reports. We report a case of a woman with uncontrolled diabetes who presented with altered mental status and was found to have a brain abscess secondary to *Lactobacillus fermentum*.

## Introduction

A brain abscess is defined as a focal infectious collection within the brain parenchyma. Bacteria can infect the brain either directly from a nearby infection (25-50% of cases) or can enter the brain parenchyma via hematogenous spread (20-35% of cases); the remainder of cases result from an unknown mechanism [1]. Presenting symptoms can vary, posing difficulty and delays in diagnosing a brain abscess. Headache was reported in 69% of patients, fevers in 53%, and focal neurologic deficits in 48%; the classic triad of fever, headache, and focal neurologic deficits is seen only in 20% of patients [1]. Mortality from brain abscesses can range from 15% to 31% [1]. Brain abscess can be caused by bacteria, mycobacteria, fungi, and parasites. The most common pathogens include Staphylococcus species, Streptococcus species, Gram-negative bacilli, and polymicrobial species from skin/oral flora [2,3]. Lactobacillus is an unusual pathogen to be isolated from a brain abscess. It is a catalase-negative, Gram-positive rod, and facultative anaerobic bacteria that can be found as a commensal organism in the gastrointestinal tract, genitourinary tract, and the oral cavity. There are rare case reports where the pathogen is isolated from brain tissue with a possible odontogenic primary source [4-6]. We present a case of a middle-aged woman with uncontrolled diabetes who had *Lactobacillus fermentum* isolated from a left parietal lobe brain abscess in the absence of bacteremia or a clear source of infection.

## Case presentation

A 63-year-old female with a 20-year history of insulin-dependent diabetes mellitus and hypertension presented to the hospital due to a change in mental status for two days. At home, the patient appeared to be confused, subsequently unable to speak coherently, and then was only mumbling words hence was brought to the emergency department. The family denied any reported symptoms of upper respiratory infections, fever, ear pain, oral pain, seizure-like activity, abdominal pain, nausea, vomiting, or diarrhea. The patient did not have any recent infections, international travel, or pets at home. They denied a history of smoking, alcohol, or illicit drug use in the patient. They did report issues with medication adherence, especially insulin.

**Figure 1:**
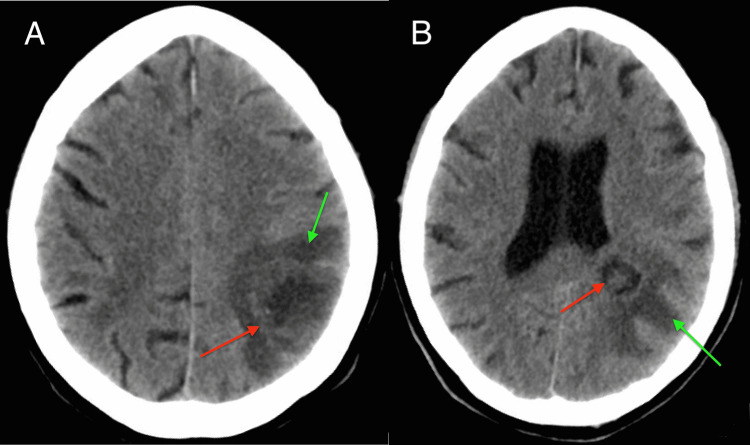
Computed tomography head without intravenous or oral contrast, axial view. Red arrows show a centrally necrotic lesion with internal heterogeneity in the left parietal lobe (A) extending towards the left lateral ventricle (B) with surrounding vasogenic edema (green arrows) and localized mass effect. It measures 3.5 x 2.5 x 2.5 cm. This is suggestive of a pyogenic or atypical abscess or a necrotic/cystic tumor.

On examination, the patient had a temperature of 100.2°F, heart rate of 100 beats per minute, blood pressure of 185/95mmHg, respiratory rate of 18 breaths per minute, and an oxygen saturation of 99% on room air. She was awake, not oriented to self, place, or time, unable to follow commands, and had aphasia. She did not have nuchal rigidity. On oral examination, there were no missing teeth or dental caries, and no inflammation of the gingiva or soft palate. There was no sinus tenderness or palpable lymphadenopathy. Her abdomen was soft, nondistended, and nontender. Laboratory investigation was significant for white blood cell count 8.4K/UL (with 88% neutrophils), hemoglobin 7.7g/dL, platelets of 712K/UL, lactic acid 1.5mmol/L, sodium 136mmol/L, potassium 4.2mmol/L, glucose 345mg/dL, bicarbonate 5mmol/L, blood urea nitrogen 8.7mg/dL, and creatinine 0.7mg/dL. Hemoglobin A1c was 18%. Serology for hepatitis B and C and HIV were non-reactive. Initial computed tomography (CT) head without contrast revealed a pyogenic or atypical abscess or a necrotic/cystic tumor in the left parietal lobe extending towards the left lateral ventricle with surrounding vasogenic edema and localized mass effect (Figures 1A, 1B).

**Figure 2:**
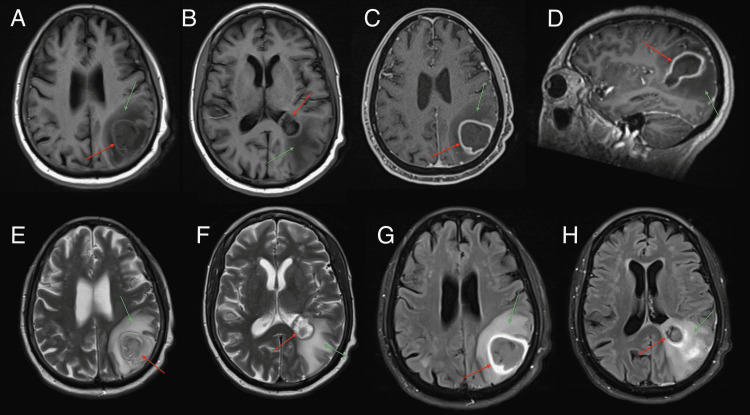
Magnetic resonance imaging of the brain with and without intravenous contrast. The images show a 3.2 X 3.2 X 3.1cm rim-enhancing left parietal lesion likely to reflect an abscess (red arrows) with vasogenic edema and mass effect (green arrows). (A and B) Both axial views are T1-weighted without contrast; (C) axial view and (D) sagittal view are T1-weighted with contrast highlighting the rim-enhancing lesion. (E and F) both axial views are T2-weighted images. (G and H) both axial views are FLAIR images. FLAIR: fluid-attenuated inversion recovery

Magnetic resonance imaging (MRI) of the brain with and without contrast was performed which showed a 3.2 x 3.2 x 3.1cm rim-enhancing left parietal lesion likely to reflect an abscess; also noted was surrounding vasogenic edema and localized mass effect (Figures 2A-2H). Additionally, the visualized paranasal sinuses, orbits, and mastoid air cells were unremarkable.

The patient had two sets of blood cultures drawn and was then started on broad-spectrum antibiotics with ceftriaxone, vancomycin, and metronidazole; she was also given levetiracetam for seizure prophylaxis. She was evaluated by neurosurgery and underwent a left craniotomy with abscess evacuation without complication. Blood cultures and urinalysis remained negative throughout the patient's hospitalization. The abscess culture was negative for acid-fast bacilli and fungi but did return positive for *Lactobacillus fermentum*, which was sensitive to penicillin and resistant to vancomycin. Thus, antibiotics were de-escalated to ampicillin. To further evaluate the source of infection, a transthoracic echocardiogram was performed and was negative for any vegetations. The patient's mental status improved greatly during her hospitalization and she was discharged on hospital day eight with an appropriate insulin regimen, levetiracetam for seizure prophylaxis, and a peripherally inserted central catheter for ampicillin infusion for at least six to eight weeks. She was also scheduled for outpatient appointments with gastroenterology for a colonoscopy and with a dentist to further rule out any odontogenic source of infection.

## Discussion

Brain abscesses are focal infectious collections within the brain parenchyma; they can result from bacteria, fungi, or parasites. If a single abscess is seen, it is typically the result of direct spread (which is the most common mechanism of development) [1]. The location of the abscess can also provide more information regarding the primary source. For example, otitis media and mastoiditis typically spread to the inferior temporal lobe and the cerebellum. Sinusitis from the frontal or ethmoid sinuses or dental infections typically spread to the frontal lobe. In contrast, if multiple abscesses are seen in the brain, it is typically the result of hematogenous spread; these lesions are usually found in the distribution of the middle cerebral artery [7].

The exact organism causing brain abscess depends on the patient's co-morbidities and the mechanism of infection. Severely immunocompromised patients, such as those with organ transplants or HIV, can have infections with tuberculosis or nonbacterial infections (fungal or parasitic) [2]. Patients who have had neurosurgery or head trauma develop infections with skin flora, such as Staphylococcus species or Gram-negative bacilli [3]. On the other hand, direct spread of infections can result in abscesses caused by streptococci and polymicrobial species [8].

*Lactobacillus fermentum* is Gram-positive rod and facultative anaerobic bacteria; it can be found as a commensal organism in the gastrointestinal tract, genitourinary tract, and the oral cavity. Uncommonly, Lactobacillus species can be pathogenic, resulting in bacteremia, endocarditis, dental abscesses, meningitis, liver abscess, and other intra-abdominal infections [9-11]. There are sparse case reports where Lactobacillus species are isolated from brain tissue, with a possible odontogenic primary source, however, these reports found *Lactobacillus catenaformis* and not *Lactobacillus fermentum*, which was isolated from our patient [4-6].

This patient had diabetes which was not well controlled, as evident by the HbA1c of 18%, which left her severely immunocompromised. The mechanism in which she developed the brain abscess was also unclear. She did not have bacteremia which would have resulted in hematogenous seeding. She also did not have a clear source of primary infection that could have spread to the brain. She did not have any head trauma or prior neurosurgeries. Her oral examination was reassuring as there was no clear evidence of an odontogenic source of infection. An abdominal source was unlikely as there were no gastrointestinal symptoms and abdominal examination was benign. All the patient's blood cultures and urinalysis were negative. Transthoracic echocardiogram did not show any vegetations suggestive of endocarditis. Finally, the MRI brain did not show any abnormalities in any of her facial sinuses to suggest sinusitis, nor did she have signs/symptoms of sinusitis. The patient could have had a dedicated dental evaluation or an abdominal CT scan to further rule out definite sources - these are limitations to the case report, especially since *Lactobacillus fermentum* has been isolated in dental caries [12,13]. Since our patient was clinically improving after drainage of the abscess and implementation of intravenous antibiotics, dental evaluation was referred to the outpatient setting. This case report presents a novel case of a brain abscess caused by *Lactobacillus fermentum*. This patient had appropriate management with source control of the abscess in addition to long-term, pathogen-directed intravenous antibiotics. Due to its low incidence, high-powered studies are needed to better understand the pathogenesis of Lactobacillus species causing brain abscesses in immunocompromised patients, such as those with diabetes.

## Conclusions

Brain abscesses can be caused by a variety of unusual organisms, particularly in patients who are immunocompromised. Due to the high mortality associated with brain abscesses, prompt recognition and treatment are critical. In addition to source control, broad-spectrum antibiotics must be implemented until culture and susceptibility data are available. Our case highlights an uncontrolled diabetic patient who had a brain abscess caused by *Lactobacillus fermentum*​​​​​​.
